# Evaluation of right ventricular functions in patients with ischemic cardiomyopathy by speckle-tracking echocardiography

**DOI:** 10.1186/s43044-024-00566-3

**Published:** 2024-09-28

**Authors:** Reham Mohamed Darweesh, Dina Mohamed Yousry Ahmed, Kamal Mahmoud Ahmed, Wafaa Anwar El-Aroussy, Abdalla Amin Elagha

**Affiliations:** 1 Faculty of Medicine - Cardiovascular Department, Cairo University, Cairo, Egypt; 2 Medical Research Institute, Alexandria University, Alexandria, Egypt

**Keywords:** Right ventricle, Ischemic cardiomyopathy, Speckle-tracking echocardiography

## Abstract

**Background:**

It is widely recognized that the right ventricle plays a significant role in the prognosis of numerous diseases. However, the assessment of right ventricular function (RV) has not been given much attention until recently. This study used speckle-tracking echocardiography (STE) to assess RV functions in ischemic cardiomyopathy (ICM) patients.

**Results:**

This study included 74 patients diagnosed with ischemic cardiomyopathy (ICM) and an ejection fraction (EF) of less than 50%. Although all the selected patients had normal RV systolic function by tricuspid annular plane systolic excursion (TAPSE), a considerable percentage of them had subtle RV systolic dysfunction, which could be identified by right ventricular free wall longitudinal strain (RV FWLS) (36.5%) and right ventricular global longitudinal strain (RV GLS) (55.4%). Moreover, the mean RV FWLS was significantly higher than RV GLS (− 20.4 ± 5.08% vs. − 17.5 ± 6.89%), respectively. Advanced left ventricle (LV) adverse remodeling was associated with subtle RV dysfunction. Using multivariate regression analysis, increased E/e' (*p* = 0.016, CI 1.135–3.423) and RV myocardial performance index (MPI) (*p* = 0.007, CI 0.000–0.007) were identified as independent factors of impaired RV FWLS with the greatest effectiveness.

**Conclusion:**

When standard RV measures are normal in patients with ICM, RV systolic strain analysis offers an incremental utility to detect subtle abnormalities in RV function, especially in resource-constrained settings where cardiac magnetic resonance (CMR) is not practical.

## Background

As per the latest European Association of Cardiovascular Imaging (EACVI) guidelines on chamber quantification, tricuspid annular plane systolic excursion (TAPSE) ≥ 17 mm, fractional area change (FAC) ≥ 35%, and lateral tricuspid annulus peak systolic velocity (S′) ≥ 9.5 cm/s are the standard RV systolic function parameters [1]. Strain is an innovative method that allows for angle-independent measurement of active myocardial deformation [2]. Previous research has shown that strain can be used to identify a small but clinically significant reduction in cardiac function [3–7]. Accordingly, when conventional RV systolic measurements (TAPSE, FAC, S') are normal, subtle RV systolic abnormalities can be identified. The healthy population’s lowest predicted cutoff absolute value for both free wall longitudinal strain (RV FWLS) and global RV strain (RV GLS) is 20% [1]. When it comes to identifying early RV impairment in patients who are typically thought to be free of RV dysfunction, the superiority of RV strain as compared to conventional RV systolic indicators (TAPSE, FAC, S') may be especially intriguing [8]. The perception of subtle RV systolic abnormalities using RV systolic strain even in the presence of normal standard measurements is not yet established [9].

## Methods

### Study population

This prospective study enrolled 74 patients diagnosed with ischemic heart disease (angiographic evidence of ≥ 50% stenosis in main and/or side branch, and/or history of previous myocardial infarction) and left ventricular systolic dysfunction (ejection fraction less than 50%) who presented to the cardiology outpatient clinic at Kasr Al-Ainy Medical School, Cairo university, from September 2019 to September 2020. This research is approved by local ethical committee of Kasr Al-Ainy Medical School, Faculty of Medicine, Cairo University (N-67-2018). Patients were diagnosed based on their medical history, clinical examination, electrocardiography, echocardiography, and, if available, coronary angiography. The inclusion criteria included patients with LV systolic dysfunction (EF less than 50%) and normal RV systolic function by TAPSE (TAPSE more than 17 mm). The study excluded patients who had unsatisfactory images, atrial fibrillation, ejection fraction equal to or greater than 50%, angiographic evidence of RCA involvement, previous CABG, as well as patients with previous evidence of RV infarction. The diagnosis of right ventricular infarction was elucidated by ST-segment elevation greater than 0.1 mV in lead V4R on a 12-lead electrocardiogram (ECG) [10].

### Clinical characteristics

All patients were subjected to comprehensive medical assessment. Collected data include BMI, blood pressure, and heart rate. Patients’ symptoms were carefully assessed including NYHA class. EGFR was calculated for all subjects.

### 6-minute walk test (6MWT)

The 6MWT has been utilized to assess patients’ functional status [11]. Only patients who agreed to participate in the test and who did not have any medical contraindications (blood pressure more than 180/100 mm Hg, as well as, resting heart rate more than 120) or limitations (old age, fatigue, weakness in the peripheral muscles) were tested. The examination took place in a long hospital hallway. At the start of the test, the blood pressure and heart rate of the patient were measured and documented. Each patient was given instructions to walk, rather than run while crossing the hallway. During the test, patients were free to stop or rest at any point, especially if they had symptoms such as severe dyspnea, chest discomfort, exhaustion, or dizziness. Patients were advised to continue after their symptoms subsided and told of the amount of time that had passed during the rest period. The patient was allowed to stop the test at any time. The patient was prompted to continue the test every two minutes and told how much time was left. The test was terminated after six minutes, the patient was seated, and the patient’s postexercise blood pressure and heart rate were recorded [12].

### Echocardiography

All of the patients underwent a full echocardiography examination according to the recommendations of the American Society of Echocardiography [1], using the Vivid q echocardiography system. Measurements of the interventricular septum, LV posterior wall thickness, LV end-diastolic as well as LV end-systolic dimensions, were taken from the parasternal long-axis view. Biplane Simpson’s rule was used to calculate LV volumes and ejection fraction [1]. Using pulsed Doppler echocardiography, the ratio of peak trans-mitral early to late diastolic flow velocity (E/A) and the deceleration time (DT) of E wave velocity were determined [13]. The average of the peak early diastolic mitral annular velocity (E') at the inferior-septal and inferior-lateral segments was determined. Consequently, the E/e' ratio was computed [14]. RV linear dimensions (mid, basal, longitudinal), the fractional area change (FAC), RV end-diastolic area (EDA), and the RV end-systolic area (ESA) were measured from the RV-focused apical 4-chamber view. RV-free wall thickness was assessed using M-mode or 2D echocardiography from the subcostal window at the end-diastole. The tricuspid annular plane systolic excursion (TAPSE) was used to assess the longitudinal RV systolic function [15]. The difference between the tricuspid closure to opening time and the pulmonary ejection time, divided by the ejection time, was used to generate the RV myocardial performance index (MPI) [1]. Systolic wave velocity S' was determined by placing the pulsed tissue Doppler sample volume at the tricuspid annulus from the apical 4-chamber view [15]. The mean right atrial pressure was determined by measuring the diameter of the inferior vena cava and its respiratory variation. Additionally, the maximal pressure difference between the right ventricle and the right atrium was determined by trans-tricuspid continuous-wave Doppler flow velocity. Using tissue Doppler of the lateral tricuspid annulus and pulsed Doppler of the tricuspid inflow, the diastolic function of the RV was evaluated [16, 17]. Diastolic indexes (E/e' ratio, E/A ratio, and deceleration time) were measured [11]. From the apical 4-chamber view, measurements were taken of the right atrial area, major and minor dimensions, and indexed left atrial volume (LAVI) [1]. The vena contracta and jet area from the apical 4-chamber image were used to grade mitral regurgitation [18] and tricuspid regurgitation [19].

### Speckle-tracking strain and strain rate analysis

**Fig. 1:**
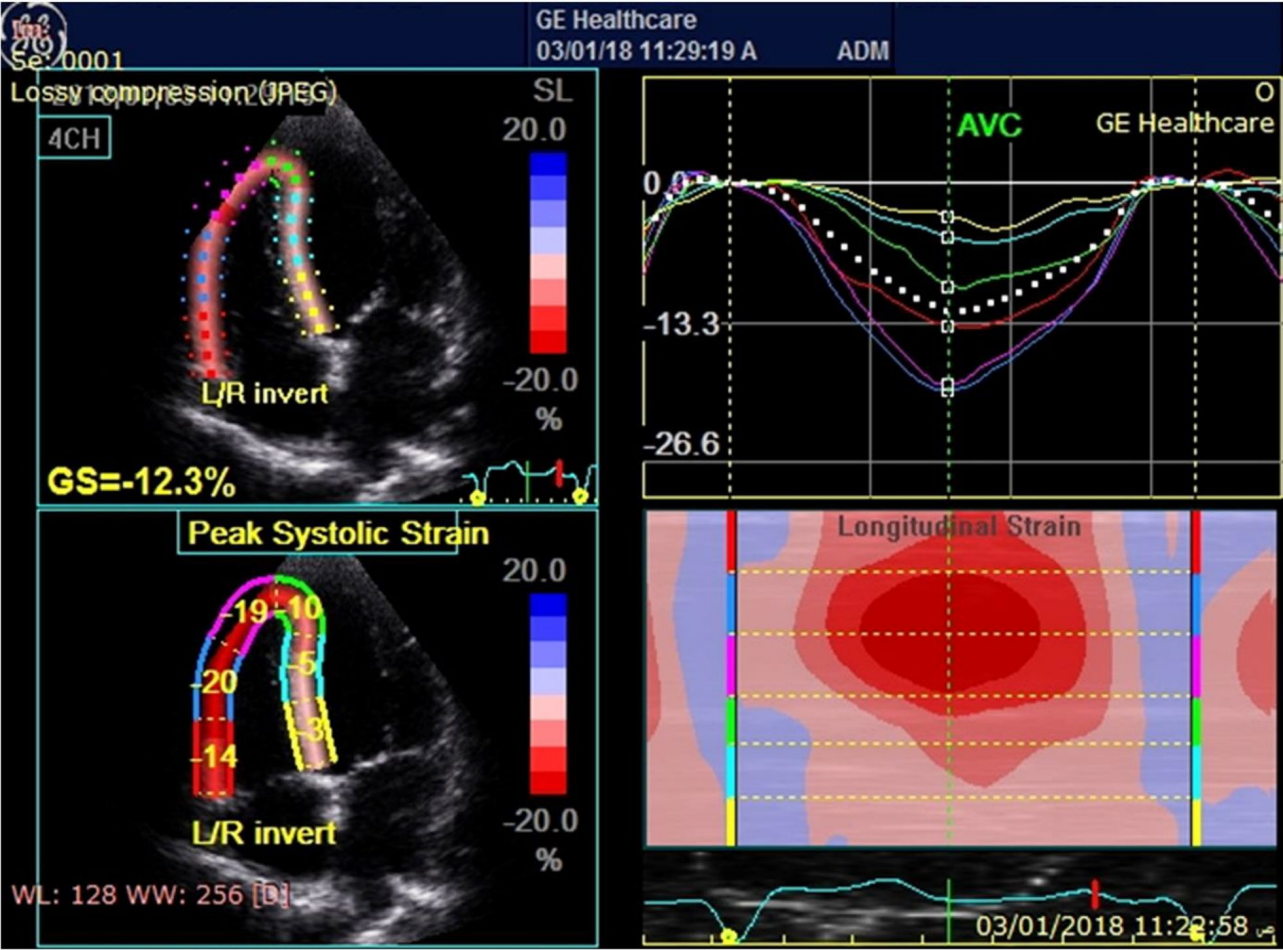
The measurement of RV GLS and RV FWLS from apical 4-chamber view. A 60-year-old male patient was considered as having normal RV function by TAPSE (2.1 cm); however, RV GLS was − 12.3% and RV FW PSS was − 17.6%

Both the LV and RV regional and global myocardial strain were calculated using speckle-tracking analysis. In the long-axis, 2-chamber, and apical 4-chamber images, the longitudinal left ventricular strain was measured. RV strain was measured longitudinally using the apical 4-chamber view [20]. The average frame rate was at least 50 Hz. With the use of specialized software, recorded images were analyzed offline (Echo Pac, version 113). The percentage change from the initial end-diastolic dimension is used to express cardiac strain. Myocardial thinning or shortening is indicated by negative values, whereas cardiac thickening or lengthening is shown by positive values [21]. The LV was then automatically divided into six segments in the apical 4-chamber, long-axis, and 2-chamber views, and the RV in the RV-focused apical 4-chamber view (Fig. 1). The indices of cardiac systolic contraction were identified as peak systolic strain (PSS), which was determined by the use of time-strain curves. The free wall longitudinal strain (RV FWLS) was determined by the average strain signals from the RV basal lateral, mid-lateral, and apical lateral segments. RV global longitudinal strain (RV GLS) was evaluated in the six segments [22]. From the strain rate curves of the six segments of the RV and LV, respectively, positive RV and LV early diastolic peak strain rate (SRe) and negative RV and LV peak systolic strain rate (RV SRs, LV SRs) were determined [23].

### Statistical methods

The statistical software for the social sciences (SPSS) version 26 (IBM Corp., Armonk, NY, USA) was used. Variables were normally distributed (using the Kolomgorov–Smirnov test). As for the quantitative variables, the mean and standard deviation were used to summarize the data; for categorical variables, the frequencies (number of cases) and relative frequencies (percentages) were used. Unpaired t-tests were used to compare the groups [24]. An analysis of categorical data was done using the Chi-square (χ2) test [25]. The Pearson correlation coefficient was performed for correlations of quantitative variables [26]. We used logistic regression to find independent parameters linked to impaired RVFWLS [27]. The selection of parameters for consideration for entry in the multivariable model was based on univariable statistical significance. Significant variables were included in the multivariable model using the Enter method. *p*-values less than 0.05 were considered statistically significant.

## Results

Out of 116 screened patients, 74 patients (mean age 56.74 ± 7.66), met eligibility criteria during the study period. The following are some of our study’s key findings: (1) in ICM patients, RV global and free wall systolic strain as well as strain rate are crucial metrics to evaluate the RV’s myocardial systolic function. (2) It is crucial to consider the incremental benefit of incorporating RV systolic strain into standard RV measures to identify subtle RV systolic abnormalities.

The following information is presented in Table 1 which displays the clinical and echocardiographic features of the people who participated in the study.

**Table 1:** Clinical and echocardiographic characteristics of the study population

		RV FWLS				*p* value	RV GLS				*p* value	Abnormality threshold^a^
		Impaired		Not impaired			Impaired		Not impaired			
		Mean	SD	Mean	SD		Mean	SD	Mean	SD		
BMI		21.31	1.70	21.20	1.75	0.787	21.49	1.70	20.93	1.72	0.167	
SBP		136.30	19.32	130.70	17.73	0.210	134.56	22.97	130.48	10.15	0.312	
DBP		81.81	13.51	78.96	9.18	0.334	82.27	12.02	77.18	8.85	0.046*	
HR		90.22	7.60	75.00	8.02	< 0.001*	86.90	8.71	72.67	7.27	< 0.001*	
6MWT		257.37	47.47	388.40	48.01	< 0.001*	292.59	65.78	400.24	48.11	< 0.001*	
EGFR		57.49	10.22	69.73	5.50	< 0.001*	60.83	10.09	70.78	4.98	< 0.001*	
LVE DVI		78.33	20.70	53.15	13.89	< 0.001*	72.49	22.22	49.73	7.25	< 0.001*	> 74 Male, > 61 Female
LVE SVI		48.04	15.57	29.51	8.02	< 0.001*	43.27	15.79	27.58	4.60	< 0.001*	> 31 Male, > 24 Female
EF %		38.93	5.01	43.74	3.10	< 0.001*	40.29	4.83	44.09	3.05	< 0.001*	< 50%
PWT		1.02	0.15	1.00	0.14	0.612	1.01	0.15	1.00	0.13	0.694	
SWT		0.97	0.18	0.92	0.13	0.193	0.95	0.16	0.92	0.14	0.416	
E cm/s		108.37	15.04	82.47	13.99	< 0.001*	102.76	16.87	78.45	11.48	< 0.001*	
é cm/s		4.30	1.97	6.16	1.78	< 0.001*	4.73	2.10	6.41	1.56	< 0.001*	
E/é		20.97	3.24	12.70	2.76	< 0.001*	18.68	4.57	12.03	2.21	< 0.001*	> 14
LAVI		71.76	13.14	41.01	9.11	< 0.001*	63.51	16.78	38.21	6.96	< 0.001*	> 34 ml/m^2^
TAPSE		2.00	0.18	2.38	0.28	< 0.001*	2.08	0.22	2.43	0.29	< 0.001*	< 17
FAC		36.74	3.53	41.72	3.35	< 0.001*	38.00	3.65	42.27	3.54	< 0.001*	< 35%
S velocity		10.55	0.87	13.41	1.78	< 0.001*	11.18	1.51	13.84	1.63	< 0.001*	< 9.5
TRPG		57.19	10.64	39.94	6.22	< 0.001*	52.39	11.67	38.58	5.26	< 0.001*	< 35
IVC		2.13	0.26	1.47	0.31	< 0.001*	1.96	0.36	1.41	0.30	< 0.001*	> 2.2 cm
RV MPI		0.40	0.11	0.74	0.12	< 0.001*	0.50	0.18	0.76	0.12	< 0.001*	> 0.54 (DTI)
RV wall thickness		1.23	0.10	0.94	0.15	< 0.001*	1.17	0.13	0.90	0.14	< 0.001*	< 5 mm
LV GLS		− 11.06	7.86	− 18.26	2.42	< 0.001*	12.94	7.00	18.98	2.08	< 0.001*	> − 20%
RVSRe		0.90	0.30	0.42	0.11	< 0.001*	0.74	0.33	0.42	0.10	< 0.001*	
RV SRs		0.68	0.28	1.06	0.18	< 0.001*	0.77	0.27	1.10	0.19	< 0.001*	
NYHA	1	0	0.0%	19	40.4%	< 0.001*	3	7.3	16	48.5	< 0.001*	
	2	7	25.9%	27	57.4%		17	41.5	17	51.5		
	3	19	70.4%	1	2.1%		20	48.8	0	0.0		
	4	1	3.7%	0	0.0%		1	2.4	0	0.0		

*6MWT* 6-min walk test, *BMI* body mass index, *DBP* diastolic blood pressure, *DTI* Doppler tissue imaging, *EF* ejection fraction, *EGFR* estimated glomerular filtration rate, *FAC* fractional area change, *HR* heart rate, *IVC* inferior vena cava, *LAVI* left atrial volume index, *LVEDVI* left ventricle end-diastolic volume index, *LVESVI* left ventricle end-systolic volume index, *LV GLS* left ventricle global longitudinal strain, *NYHA* New York heart association, *PWT* posterior wall thickness, *RV FWLS* right ventricle free wall longitudinal strain, *RV GLS* right ventricle global longitudinal strain, *RV MPI* right ventricle myocardial performance index, *RV SRs* right ventricle systolic strain rate, *RV SRe* right ventricle early diastolic strain rate, *S* systolic wave velocity, *SBP* systolic blood pressure, *SWT* septal wall thickness, *TAPSE* tricuspid annular plane systolic excursion, *TR PG* tricuspid regurgitation peak gradient

*Statistically significant at *p* ≤ 0.05

^a^according to ASE recommendations for chamber quantification [1]

### The utility of RV systolic stain to identify subtle RV dysfunction

Using RV FWLS and RV GLS in place of traditional measures (TAPSE, S', and FAC), subtle RV systolic abnormalities were detected. RV systolic strain identified subtle RV dysfunction in an enormous proportion of patients, even though all the patients chosen had normal RV longitudinal systolic function based on TAPSE (> 17 mm). Of these, 27 (36.5%) were diagnosed with RV dysfunction based on RV FWLS (> − 20%), and 41 patients (55.4%) based on RV GLS (> − 20%). Additionally, the mean RVFWLS was higher in those patients than RV GLS (− 20.4 ± 5.08% vs. − 17.5 ± 6.89%), which may be a result of the bias of including the septal longitudinal strain values. Normal RV function by the conventional parameters (TAPSE, S', FAC) was in 54 patients out of 74 total subjects. Of these 13 (24%) patients with normal all conventional parameters, had abnormal RV strain results.

Table 2 presents a univariate analysis of associations for impaired RV function using RV systolic strain. Heart rate, NYHA class, the shorter walking distance at 6 MWT, and lower GFR were related to the occurrence of subtle RV dysfunction. Patients with subtle RV dysfunction had more advanced adverse remodeling as measured by LV dimensions (larger EDV, ESV) or LV functions (systolic; lower EF, diastolic; E, e', E/e') and larger LAVI. RV hypertrophy (increased free wall thickness), dilated IVC, and higher TR PG are all associations of RV dysfunction measured by RV systolic strain.

**Table 2:** Univariate analysis of associations of RV dysfunction

	RV FWLS		RV GLS-T		*N*
	*r*	*p* value	*r*	*p* value	
RV GLS-T	0.643	< 0.001*			74
AGE	− 0.348	0.002*	− 0.130	0.269	74
BMI	− 0.134	0.256	− 0.080	0.500	74
SBP	− 0.077	0.517	− 0.046	0.697	74
DBP	− 0.085	0.471	− 0.012	0.920	74
HR	− 0.686	< 0.001*	− 0.537	< 0.001*	74
6MWT	0.853	< 0.001*	0.562	< 0.001*	74
EGFR	0.716	< 0.001*	0.413	< 0.001*	74
LVEDVI	− 0.665	< 0.001*	− 0.344	0.003*	74
LVESVI	− 0.707	< 0.001*	− 0.346	0.003*	74
EF %	0.550	< 0.001*	0.230	0.049*	74
PWT	− 0.059	0.619	− 0.125	0.289	74
SWT	− 0.157	0.181	− 0.168	0.153	74
E cm/s	− 0.698	< 0.001*	− 0.448	< 0.001*	74
é cm/s	0.463	< 0.001*	0.303	0.009*	74
E/é	− 0.805	< 0.001*	− 0.527	< 0.001*	74
LAVI	− 0.858	< 0.001*	− 0.556	< 0.001*	74
TAPSE	0.657	< 0.001*	0.469	< 0.001*	74
FAC	0.683	< 0.001*	0.456	< 0.001*	74
S velocity	0.720	< 0.001*	0.456	< 0.001*	74
TRPG	− 0.739	< 0.001*	− 0.399	< 0.001*	74
IVC	− 0.800	< 0.001*	− 0.453	< 0.001*	74
RVMPI	0.860	< 0.001*	0.580	< 0.001*	74
RV wall thickness	− 0.766	< 0.001*	− 0.457	< 0.001*	74
LV GLS	0.530	< 0.001*	0.606	< 0.001*	74
RVSRe	− 0.763	< 0.001*	− 0.550	< 0.001*	74
RV SRs	0.614	< 0.001*	0.625	< 0.001*	74
NYHA	− 0.723	< 0.001*	− 0.552	< 0.001*	74

*r*: Pearson coefficient

*6MWT* 6-min walk test, *BMI* body mass index, *DBP* diastolic blood pressure, *EF* ejection fraction, *EGFR* estimated glomerular filtration rate, *FAC* fractional area change, *HR* heart rate, *IVC* inferior vena cava, *LAVI* left atrial volume index, *LVEDVI* left ventricle end-diastolic volume index, *LVESVI* left ventricle end-systolic volume index, *LV GLS* left ventricle global longitudinal strain, *NYHA* New York heart association, *PWT* posterior wall thickness, *RV FWLS* right ventricle free wall longitudinal strain, *RV GLS* right ventricle global longitudinal strain, *RV MPI* right ventricle myocardial performance index, *RV SRs* right ventricle systolic strain rate, *RV SRe* right ventricle early diastolic strain rate, *S* systolic wave velocity, *SBP* systolic blood pressure, *SWT* septal wall thickness, *TAPSE* tricuspid annular plane systolic excursion, *TR PG* tricuspid regurgitation peak gradient

*Statistically significant at *p* ≤ 0.05

The correlations between RV FWLS and age (*r* − 0.348, *p* 0.002), heart rate (*r* − 0.686, *p* 0.001), GFR (*r* 0.716, *p* 0.001), and NYHA class (*r* − 0.723, *p* 0.001) were statistically significant.

### 6-min walk test

A statistically significant correlation between the distance walked during 6 MWT and RV FWLS (*r* 0.853, *p* < 0.001) was found.

### RV systolic function

**Fig. 2:**
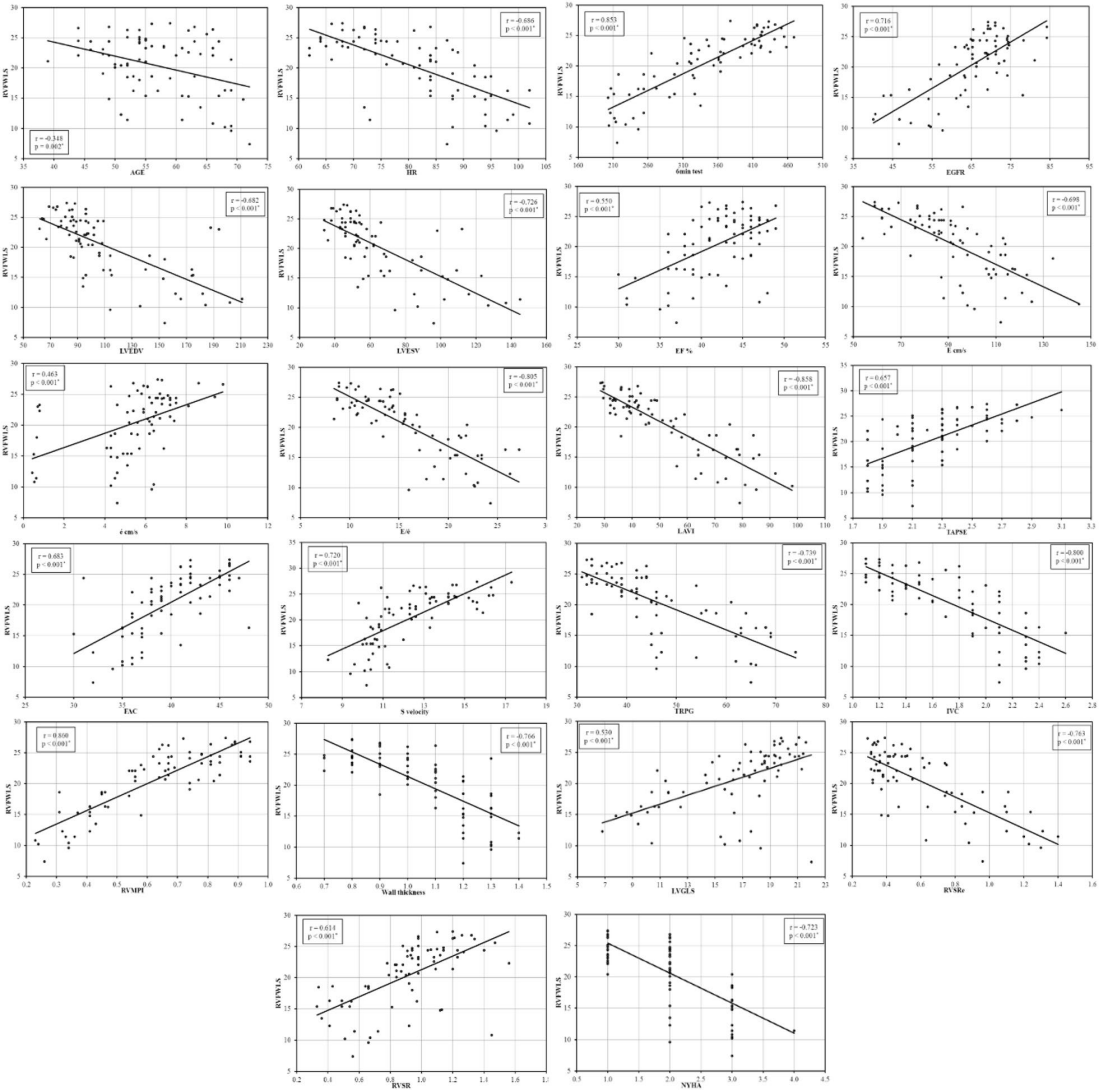
Scatter plots of correlations of RV FWLS to different clinical and echocardiographic parameters

Table 2 presents a statistically significant relationship (with all *p*-values < 0.001) between RV FWLS and conventional RV indicators, such as RV MPI, FAC, TAPSE, and S' velocity. Additionally, as shown in Fig. 2, there is also a statistically significant association between RV FWLS and RV SRs (*p*-value < 0.001).

### RV diastolic function

RV SRe and RV FWLS exhibited a statistically significant negative association (*r* − 0.763, *p* < 0.001) for RV diastolic function (Fig. 2).

### Other parameters related to RV

There was a statistically significant negative correlation between TR PG (*r* − 0.739, *p* < 0.001), IVC diameter (*r* − 0.800, *p* < 0.001), RV-free wall thickness (*r* − 0.766, *p* < 0.001), and RVFWLS.

### Associations of RV FWLS with LV function parameters

A statistically significant association was seen between LV GLS, EF, and RV FW LS concerning LV systolic function (*r* 0.530, *p* < 0.001; *r* 0.550, *p* < 0.001, respectively).

There was a statistically significant association (*r* − 0.805, *p* < 0.001) between LV E/e' and RV FWLS concerning LV diastolic function. Additionally, a statistically significant negative association was found between RV FWLS and LV volumes (LVEDVI: *r* − 0.665, *p* < 0.001; LVESVI: *r* − 0.707, *p* < 0.001), as well as between LAVI and RV FWLS (*r* − 0.858, *p* < 0.001) (Fig. 2).

### RV GLS versus RV FWLS

**Fig. 3:**
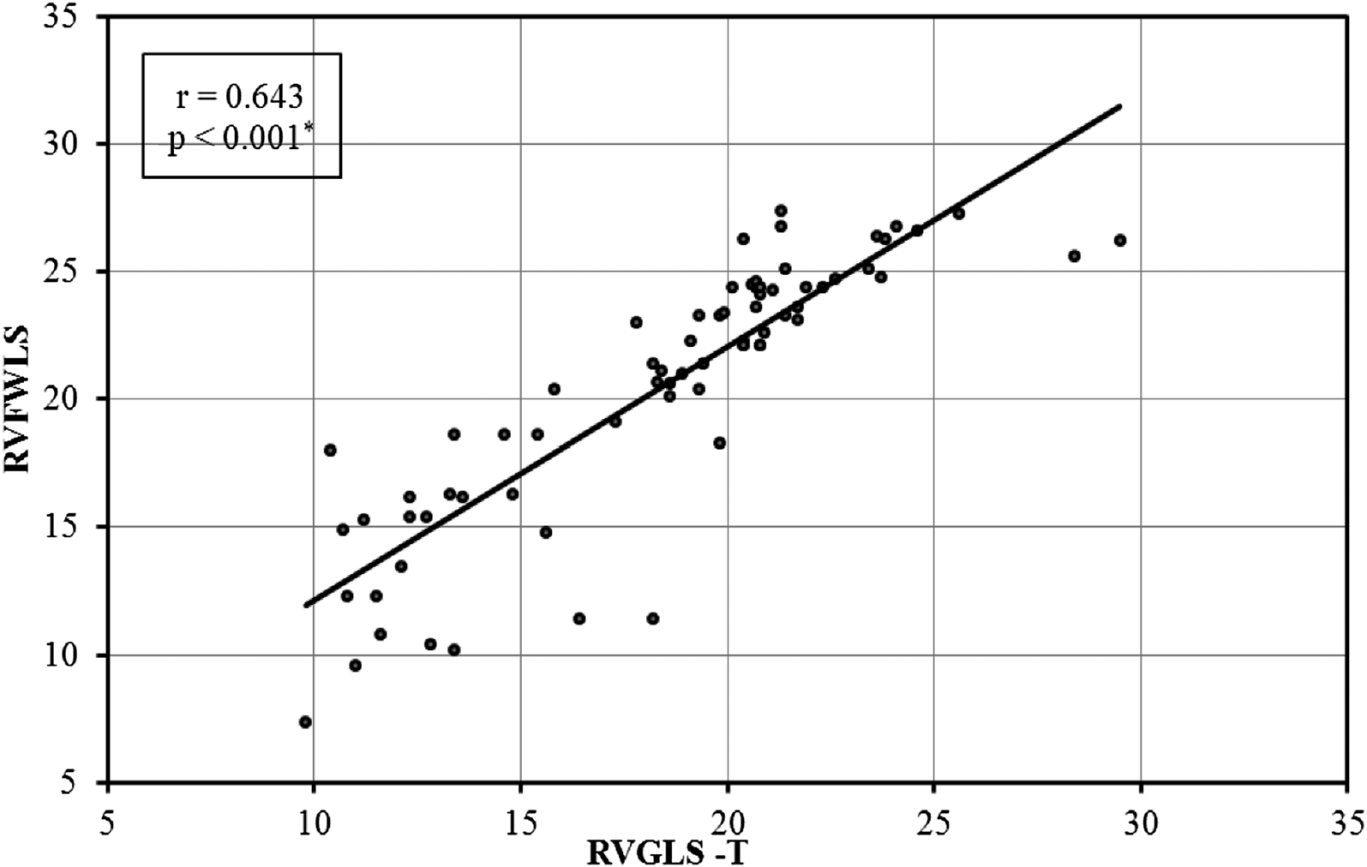
Correlation between RV GLS and RV FWLS

As shown in Fig. 3, there was a significant correlation between RVFWLS and RV GLS (*p* < 0.001).

Using multivariate regression analysis of correlated conventional echocardiographic parameters, Increased E/e' (*p* = 0.016, OR 1.971, CI 1.135–3.423) and RV MPI (*p* = 0.007, OR 0.000, CI 0.000–0.007) were the only two significant associations of impaired RVFWLS while reduced S' Velocity (*p* = 0.043, OR 1.66, CI 1.016–2.725) was the most significant association of reduced RV GLS (Table 3).

**Table 3:** Multivariate logistic regression to detect independent predictors of impaired RV function

		*p* value	OR	95% CI	
				Lower	Upper
Impaired RVFWLS	E/é	0.016	1.971	1.135	3.423
	RV MPI	0.007	0.000	0.000	0.007
Impaired RV GLS -T	S velocity	0.043	1.664	1.016	2.725

*p-value* probability value, *CI* confidence intervals, *RV FWLS* right ventricle free wall longitudinal strain, *RV GLS* right ventricle global longitudinal strain

### Inter- and intra-observer variability

Many studies have confirmed the reproducibility of RV strain analysis. We analyzed the interobserver variability on 16 subjects who were randomly selected, both RV GLS and RV FWLS had low intra- (Intra class coefficient of RV GLS and RV FWLS; 0.998 and 0.997, respectively) and interobserver variability (Intra class coefficient of RV GLS and RV FWLS; 0.999 and 0.992, respectively) with absolute differences less than 1% (RV GLS 17.5 ± 6.89% and 16.8 ± 5.65%; RFWLS 20.4 ± 5.08% and 21.5 ± 4.61%, respectively).

## Discussion

In line with our research, Guendouz et al. [28] showed that RV GLS was more predictive of adverse outcomes versus conventional echocardiography markers. Additionally, Morris et al. [9] examined a cohort of 642 heart failure patients from ten centers. They observed that even in individuals with normal TAPSE, S', and FAC, subtle RV longitudinal systolic abnormalities could be disclosed by RV global and free wall systolic strain in a considerable fraction of HFrEF patients.

An interesting study involving 27 patients with HFrEF (ejection fraction < 25%) revealed a strong correlation between RV-free wall longitudinal strain and myocardial fibrosis as determined by histopathological examination. RV FWLS played a decisive part in identifying significant myocardial fibrosis [29].

Unlike our research, Fine et al. [30] in a research conducted on 116 patients without cardiopulmonary disease or risk factors, discovered a moderate association between RV strain and S' (*r* = 0.61, *p* = 0.007) as well as TAPSE (*r* = 0.58, *p* = 0.037). Previous research has shown similarly moderate correlations, probably because these factors reflect the basal function of the RV [31, 32]. In contrast to TAPSE and S′, RV strain determines for a larger area of RV-free wall deformation and is not subject to translational motion-related errors.

Additionally, Iacoviello et al. [33] found that RV GLS and RV FWLS were strongly linked with RV FAC, TAPSE, and S' velocity; the standard indices of RV systolic function. They discovered that measures indicating LV systolic function were correlated with both RV GLS and RV FWLS, which is similar to what we reported in our study. On the other hand, RV FWLS showed weaker relationships with LV EF and LV GLS than did RV GLS.

RV-free wall segments alone or the average of the 6 segments of RV-free wall and interventricular septal in the apical 4-chamber view were previously referred to as RV GLS. Yet, the two approaches produce very different results [2].

A similar study, which examined the effectiveness of speckle tracking in the evaluation of RV function in post-MI patients treated with PCI, only reported RVFWLS [2] based on the consideration that the interventricular septum is mostly a left ventricular component. Since longitudinal shortening of the RV-free wall generates most of the RV stroke volume, RV FWLS, which measures the maximal shortening in the RV-free wall from apex to base, is thought as a good reference of RV function [34]. However, to a lesser extent, the interventricular septum also plays a role in RV systolic function. The RV may benefit from the simultaneous LV systolic contraction because its workload is much less than the LV’s. In healthy hearts, LV contraction accounts for 20% to 40% of the increase in RV pressure. Because of this, interventricular septum hypo-contractility caused by LV failure will undoubtedly contribute to RV functional impairment [35].

Iacoviello et al. [33] discovered, in line with our investigation, that RV GLS was significantly correlated with RV FWLS (*r*: 0.644; *p* < 0.001). RV FWLS mean values were substantially higher than RV GLS mean values (*p* < 0.001). The fact that the GLS measures the hypo-contractile septum may help to explain this. Taking into consideration the negative unit of strain, a positive correlation means that the larger the correlated parameter, the worse the assessed 2D strain right ventricular function and vice versa.

Lu et al. [36] investigated the variables linked to RV dysfunction. As opposed to our research, they discovered that RV-free wall strain and RV septal wall strain were not as significantly correlated or predictive of RV dysfunction as RV GLS. The only measure that predicted RV dysfunction was RV GLS.

Another recent study by Smolarek et al. [37] explored the usefulness of using RV strain in patients with acute ischemic injury and left ventricular systolic dysfunction (ejection fraction ≤ 45%). RV strain proved to be a valuable tool to detect subtle RV involvement even in patients successfully treated by primary percutaneous coronary intervention (PPCI). A significant change in RV FWLS was reported in the acute coronary syndrome subgroup at the time of hospitalization and at follow-up (0.62, *p* < 0.001).

RV SR measurement is still challenging even though it is currently possible and could be useful in the assessment of ischemic cardiomyopathy. As far as we are aware, this is the first investigation into the significance of RV early diastolic strain rate in individuals with ischemic cardiomyopathy. Early diastolic SR can distinguish between intact segments and at-risk segments in animal models and could detect at-risk segments even though systolic deformation was near-normal [38].

Measurements of left ventricular diastolic function by speckle-tracking strain rates can be very useful to delineate subtle LV dysfunction that was not detected by standard echocardiographic measurements as EF, according to a study by Okada et al. [39] on patients with DCM. According to experimental research, SR measurement can be a useful marker for ischemia identification. SRe of 0.8/s (sensitivity 75%, specificity 63%) was suggested as an acceptable cutoff for ischemia diagnosis based on human findings acquired during angioplasty [40].

### Study limitations

Some limitations of this study should be considered. The sample size was small, and the lack of follow-up to evaluate the predictive significance of RV GLS, RV FWLS, or RV SRe is among the study’s limitations. RV FWLS and RV GLS have a good connection with RV EF by CMR, as shown by several earlier investigations [36, 41–46]. It was noteworthy, therefore, that due to limited feasibility, we did not compare the strain assessments obtained from echocardiography with those obtained from cardiac MRI. Lastly, it is critical to remember that the 2D STE program used to analyze the RV is also used to assess the LV [23].

## Conclusion

RV’s systolic function can be generally evaluated by TAPSE, DTI S', and FAC. When CMR is not practical, RV 2D longitudinal STE strain appears to be more decisive in diagnosing mild RV systolic failure in ICM patients. Shortly, 2D strain may become the technique of choice to diagnose subtle RV systolic impairment, despite its current limited use in clinical practice.

## Data Availability

All data and materials are available.
